# FT-IR-based method for rutin, quercetin and quercitrin quantification in different buckwheat (*Fagopyrum*) species

**DOI:** 10.1038/s41598-017-07665-z

**Published:** 2017-08-03

**Authors:** Meta Kokalj Ladan, Janka Straus, Eva Tavčar Benković, Samo Kreft

**Affiliations:** Faculty of Pharmacy, University of Ljubljana, Aškerčeva cesta 7, 1000 Ljubljana, Slovenia

## Abstract

The present study explores an alternative method for antioxidants determination in buckwheat (*Fagopyrum*) samples. Buckwheat contains different amounts of the antioxidants rutin, quercetin and quercitrin in different plant parts. Buckwheat seeds are most commonly used as food; however, preparations from the herb can also be used as a rich source of rutin. Infrared spectroscopy was used for individual and sum quantification of rutin, quercetin and quercitrin in whole and ground flowers and leaves of seven different buckwheat species. Correlation coefficients *R* of calibration and independent validation set for rutin, quercetin and quercitrin were 1.00 and 0.98, 0.94 and 0.99, 0.99 and 0.95, respectively. Some of the developed models had accuracy comparable to the reference HPLC method. Additionally many different parameters that give an important insight into the FTIR technique are discussed (different plant parts, whole and ground untreated samples, 3 different resolutions, 7 spectra pre-treatments, using individual or averaged spectra, reducing spectral data input, considering additional non-spectral data). The implemented technique used no sample preparation, is non-destructive and uses very little amounts of sample. Result show that infrared spectroscopy can be a fast and environmentally friendly alternative technique for routine analysis of main flavonoids in aerial parts of buckwheat.

## Introduction

Healthy food is one of the most popular topics today. Science can barely follow the speed at which new claims are being made in less scientifically based reports. Buckwheat seeds are commonly used as food and are becoming an increasingly popular healthy food all over the world. They are a gluten-free source of carbohydrates and are an important part of the diet of patients with celiac disease. Buckwheat is considered to be a major source of the flavonoid rutin, for which antioxidant activity has been well reported^1, 2^. Flavonoids found in buckwheat also show many therapeutic effects that have been studied both *in vivo* and in clinical experiments^3, 4^.

The most common use of buckwheat is of its seeds; however, its flowering parts can also be used in the form of teas or other plant extracts. The flowering parts of buckwheat contain an even larger concentration of flavonoids, antioxidants, for which health benefits are claimed^1^. Studies show that the flowering parts of buckwheat contain high concentration of flavonoids, mainly rutin and smaller concentrations of quercetin and quercitrin^1, 5^, this was also confirmed in our analyses. Preparations made from buckwheat herb are a rich source of antioxidants, and the main contribution to the antioxidative activity is due to rutin^1, 2^.

The content of flavonoids differs significantly among not only different plant parts but also depending on the species and variety of buckwheat and more importantly the time of harvest and growth conditions, for which the data are difficult to acquire for finished products^1, 5, 6^. Therefore, there is a need for a fast and automated technique to assess the content of these important constituents in buckwheat products.

Analytical methods used for quantitative analysis of plant material are based on separation techniques; the most common are gas chromatography, liquid chromatography and capillary electrophoresis. The method for quantitative flavonoid analysis that is by far the most commonly used is high-performance liquid chromatography (HPLC). HPLC can be used for a wide range of different samples and types of analyses; it is accurate, specific and has good repeatability. The drawback of this otherwise very useful analytical techniques are time consumption and organic solvent usage for preparation of extracts of plant material which can take up to several hours^7–9^. The analysis of flavonoids takes approximately 35 minutes and uses organic solvents^7, 9^. Many steps of the analysis can be automated; however there is still a need of highly trained personnel to manage.

The number of studies demonstrating the use of infrared spectroscopy in combination with chemometrics for analyses of complex samples has grown dramatically. The main advantages are its speed, environmental friendliness, price, and possibility of in-line automatization since no or easy sample preparation is needed, and spectral acquisition is simple and fast once a good chemometric model has been developed^7, 10, 11^. However for the development of the chemometric model a highly trained personnel and large set of samples analysed by reference method are needed. Infrared spectroscopy can be used in the pharmaceutical and food industries for quality control and process monitoring. Fourier transform mid infrared spectroscopy (FT-IR) and near infrared spectroscopy (NIR) have been used for the determination of antioxidants in diverse plant samples, such as grains, fruits, oils, herbs, coffee, tea and honey^12, 13^. FT-IR has been used to determine flavonoids in different plant leaf samples^10^ and quercetin glycosides in onion (*Allium cepa*)^7^. Attenuated total reflectance technique (ATR) FT-IR has been used to determine different quercetin glycosides in onion, and models comparable to HPLC have been developed. The method was developed for predicting different groups of flavonols not individual quercetin glycosides. The samples used in the study were fresh onions and needed additional manipulation. The many steps used for preparing the extract of the sample have not been overpassed, making the method difficult and time consuming. Good correlation coefficients were obtained for calibration set and statistical analysis of validation samples provided similar parameter values for FTIR and HPLC data. However validation samples were not included in correlation analysis and the accuracy of the method for these samples is not evident^7^. A similar ATR technique has been used to determine antioxidants in different varieties of colored rice. Good models, with correlation coefficients *R* > 0.9, have been obtained for antioxidant activity and phenolic compounds content. The study analysed total antioxidant capacity and different groups of antioxidants and not specific compounds^14^. Only NIR has been used to determine some components of buckwheat. NIR has been used to determine the content of moisture, fat, protein, starch, amylose, and tannins in buckwheat flours. Good models were obtained for main constituents; moisture, protein and starch, with correlation coefficients 0.93, 0.90 and 0.84, respectively. For amylose, which is one of the two components of starch, and tannins that are present in smaller concentrations, correlation coefficients of the models were less than 0.7^8^. Another study using NIR has been used to determine single compounds such as rutin and D-*chiro*-inositol content in Tartary buckwheat (*Fagopyrum tataricum*). Good models with correlation coefficients *R* = 0.87 for rutin and *R* = 0.93 for D-*chiro*-inositol were obtained. The study used diffuse reflectance technique which is non-destructive but larger quantities of sample are needed, in their example 15 g of powdered sample was used^9^. The absorption bands of the chemical components are primarily in the mid region of infrared spectra; the absorption bands in the NIR are the overtones of the fundamental bands and are consequently weaker. Therefore, the FT-IR technique was chosen to analyse the flavonoids rutin, quercetin and quercitrin individually in the aerial parts of seven different buckwheat species. Rutin, quercetin and quercitin are very similar flavonoids with the same flavonol structure and they differ only in bound sugar units. Similar chemical entities are difficult to distinguish with infrared spectroscopy in complex plant samples. None of the revised literature has managed to separate them all. The aerial parts of buckwheat have not yet been analysed by IR techniques. The method using ATR accessory can be implemented using very little amounts of sample, approximately one squared millimetre of dried leaf to cover the diamond crystal, there is no destruction of the sample. No pre-treatment of dried whole or powdered plant material is needed which makes the method very fast. The well-established HPLC method demands preparation of extracts, which include sample grinding, long maceration (24 h) and filtration. Depending on resolution and number of scans, IR spectra are collected in approximately one minute. The goal of our work was to obtain models that give predictions comparable to HPLC using a simple and fast ATR FT-IR spectroscopy technique.

## Results and Discusion

All 6912 models were evaluated based on RMSE values. Models with both RMSEC and RMSEV values less than 3 × SD of the HPLC data and a coefficient *R* > 0.9 were evaluated as useful, and 186 models met these criteria. If additionally the RMSEV was less than 1 x SD of the HPLC data, the method was evaluated as being comparable to HPLC. 6 such models were obtained and are presented in Table 1.

**Table 1:** Parameters of 6 models with RMSEV comparable to the SD of the HPLC data for quercetin (QE) and the sum of flavonoids (SUM), and of the best models for rutin (RUT) and quercitrin (QI) that are presented in Fig. 1. All these models were obtained on flower samples.

Favonoid	Sample type	Resolution	Spectra used	Spectra pretreatment	Reduction of spectral data	Additional input data	Latent factors	*R* calibration	*R* validation	RMSEC	RMSEV
QE	ground	16	averaged	derivative	RS3	no	3	0.91	1.00	0.30	0.09
QE	ground	16	averaged	WA + deriv.	RS3	yes	3	0.94	0.99	0.25	0.06
QE	ground	16	averaged	WA + deriv.	RS3	no	3	0.94	0.99	0.26	0.07
SUM	ground	16	averaged	WD	RS2	yes	6	1.00	0.99	1.98	4.90
SUM	ground	4	separate	WA	RS3	yes	11	1.00	0.98	0.77	4.80
SUM	ground	4	separate	WA	RS3	no	11	1.00	0.98	0.77	4.86
RUT	ground	4	separate	WA	RS3	yes	8	1,00	0,98	2.19	3.63
QI	whole	4	averaged	WA + deriv.	RS1	no	3	0.99	0.95	1.13	2.48

The 6 models that are comparable to HPLC were obtained using spectra of flowers. Only three among the 186 useful models were obtained using leaf samples, and three useful models were built using joint data from leaves and flowers. Consequently, most of the models discussed further, were built on the spectra of flowers.

### Flavonoids

**Figure 1:**
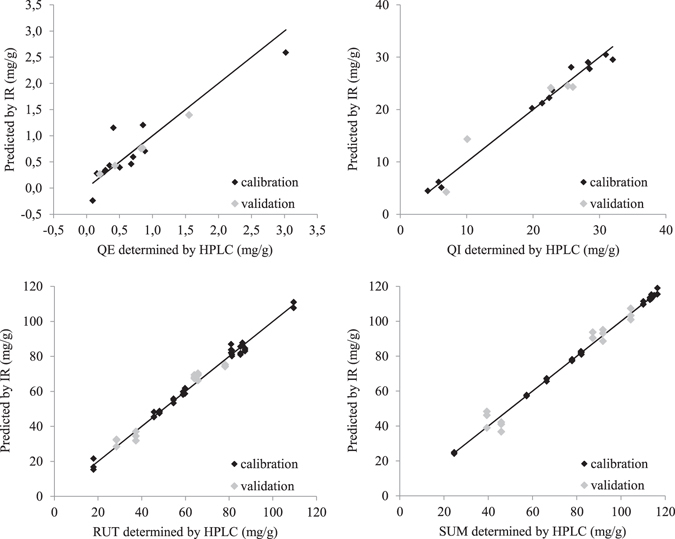
Concentrations of calibration and validation samples predicted by the best obtained models; quercetin (QE), quercitrin (QI), rutin (RUT) and the sum of flavonoids (SUM).

Models comparable to HPLC were obtained only for quercetin and the sum of flavonoids. Useful models were obtained also for rutin, but all models for quercitrin were worse than our preset limits for useful models. The best results for all four are presented in Fig. 1. Quercetin has a flavonoid aglycone structure, rutin is a quercetin glycoside with rhamnose and glucose and quercitrin is a quercetin glycoside with rhamnose. The quercetin concentration in samples was low; the flower samples contained less than 3 mg/g. Additionally, the distribution in the samples was not even; only two samples contained more than 1 mg/g. The value of 1 SD of the HPLC data was 0.1 mg/g, which was not very small compared to most of the concentrations of the samples. The preset limit for RMSE is therefore harder to exceed. The concentrations of quercitrin were below 32 mg/g, and the samples were not evenly distributed, with the majority of the samples having concentrations between 20 and 32 mg/g. The value of 1 SD of the HPLC data for quercitrin was 0.8 mg/g, which is rather small compared to most of the concentrations of the samples. The preset limit for RMSE is therefore easily exceeded (Table 2).

**Table 2:** Flavonoid concentration ranges and SD of the reference HPLC data for quercetin (QE), quercitrin (QI), rutin (RUT) and the sum of the flavonoids (SUM).

Plant part	Flavonoid	Range (mg/g)	SD_HPLC_
Flowers	QE	0.09–3	0.1
	QI	4.1–32	0.8
	RUT	18–110	3
	SUM	25–116	5
Leaves	QE	0–1.8	0.04
	QI	0–22	0.3
	RUT	11–112	3
	SUM	17–112	4
All samples	QE	0–3	0.4
	QI	0–32	0.06
	RUT	2.4–112	3
	SUM	2.8–116	4

The results show that the concentration of quercetin can be predicted from spectra despite its low concentrations and the presence of rutin and quercitrin. Therefore, the easiest available information from the spectra is of the common flavonoid structure since it can be differentiated from the structures with bound sugars. Since a good model for quercitrin (bound rhamnose) could not be obtained but a usable model for rutin (bound glucose and rhamnose) was, we suggest that the technique cannot isolate information about rhamnose bound directly to the quercetin structure very well but can differentiate if there is an additional glucose present. It is also possible that the model includes the structures with bound sugars in the same group, which contains mostly rutin, and the absolute error is therefore much smaller for rutin compared to quercitrin. Additionally, the samples were not evenly distributed in the quercitrin concentration range, which can make the building of a model more difficult.

We cannot clearly conclude whether the main contribution to different qualities of the models is different concentration range, statistical properties of data, or chemical features. Different sets of samples, with more evenly distributed concentrations, should be used to get a better insight to this problem.

From the antioxidant point of view, the most important of the parameters is the sum of flavonoid concentrations, for which a good model was obtained.

### Sample preparation

Ground and whole samples were used. There were 144 models built on spectra of ground samples and 42 on spectra of whole samples that met the criteria for useful models. All 6 models that gave comparable results to the HPLC method were built using ground sample spectra. When collecting the spectra from whole samples, the spectrum is collected from only a small surface area and therefore does not represent the average spectra of the whole sample. In the HPLC method, the analysis of the prepared extract gives a concentration that more closely resembles the average concentration of the sample. This type of information is more likely to be captured in the spectra of ground samples.

### Resolution

Different resolutions for spectra collection were used. Useful models were obtained using all three resolutions, including 66 models based on spectra collected at 4 cm^−1^, 65 based on spectra collected at 8 cm^−1^, and 55 models based on spectra collected at 16 cm^−1^. Most of the models that have a comparable RMSEV to the average SD of the HPLC data were collected at 16 cm^−1^, but some were collected at 4 cm^−1^. Therefore, it is difficult to conclude which resolution is in general most appropriate. Measuring the spectra at a lower resolution, 16 cm^−1^, is faster, but an appropriate spectral pretreatment technique must be applied to highlight the information, which can be seen from the results comparing the different pretreatment techniques. In previous studies of differentiation among plant species, it has been discovered that the detailed structures of the spectra collected at high resolution contain important information^15^. This could imply that more detailed information of overlapping bands of chemical compounds is more important for plant species differentiation compared to quantitative studies.

### Averaging the spectra

**Figure 2:**
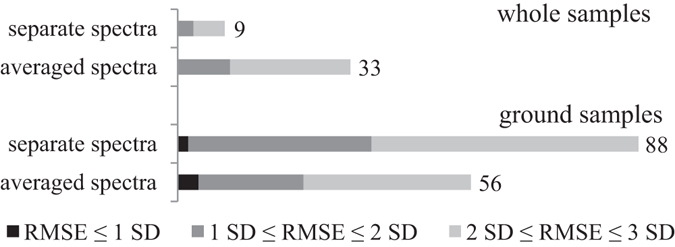
Number of models obtained with all three spectra or averaged spectra from whole and ground samples. The numbers next to the columns represent the total number of obtained useful models.

For each sample, three spectra were collected. Models were built separately on data that contained these three spectra individually and on data containing only the averaged spectra of each sample. Using whole samples, better results were obtained from the averaged spectra (Fig. 2). For the whole samples, each spectrum is from one small area, and averaging the three spectra better represents the average of the sample, which is more comparable to the concentration obtained with the HPLC method. Using ground samples, more useful models were obtained when using all three spectra, which contain more information about the sample; therefore, it is easier to recognize noise. However, more models that are comparable to the HPLC technique were obtained on averaged spectra of ground samples.

### Spectral data pretreatment

**Figure 3:**
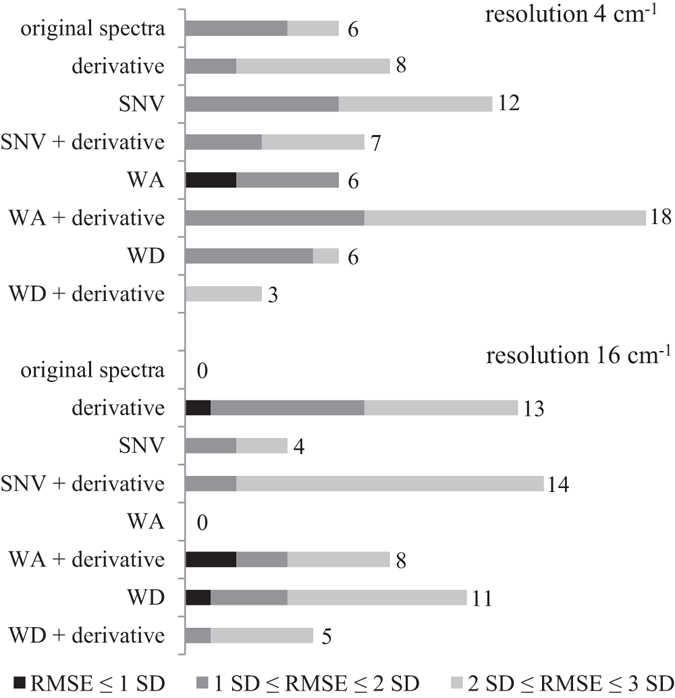
Number of models obtained with different pretreatment techniques and measured at two different resolutions. The numbers next to the columns represent the total number of obtained useful models.

Different methods for data pretreatment were applied. In our previous work, it was shown that smoothing techniques can cause a loss of important data^15^. The pretreatment techniques used are standard normal variate transform (SNV) and wavelet transform, using approximate (WA) and detailed (WD) coefficients separately. These techniques and the original spectra were also used in combination with the first derivative. The results show that derivatives in general improve the models except in combination with WD (Fig. 3). The improvement depends on the resolution. It is less apparent with the higher resolution (4 cm^−1^) and most apparent with the lowest of the used resolutions (16 cm^−1^). Our previous research showed that too much smoothing is not appropriate for spectra of multi-component samples on which complex chemometric models are built^15^. Here, we wanted to improve this knowledge by comparing different resolutions in combination with different pretreatment techniques. The derivative is a technique that emphasizes detailed structures in the spectrum, which can also be noise. At lower resolution, there is less noise, and the derivative is therefore very useful. More interesting is that the basic spectra are more useful at higher resolution, which implies that this information is somehow lost in the lower resolution such that it can be obtained only by using the derivative. However, in previous studies of differentiation among different plant species, the derivative was also useful in higher resolutions^15^. The best models were obtained at resolutions of 4 cm^−1^ and 16 cm^−1^. In the higher resolution, the approximate part of the wavelet transform gave the two best models. However, the studies of plant species differentiation showed that the detailed coefficients of the wavelet transform are also important. With lower resolution, the derivatives of basic spectra, the derivative of approximate wavelet transform, and detailed wavelet transform gave the best models. This implies that important information is present in spectra recorded at 4 cm^−1^ together with some noise. In the basic spectra recorded at 16 cm^−1^, the important information is lost and can be obtained by the derivative and wavelet transform detailed coefficients. The derivative of the detailed wavelet transform gives worse results, implying that these data contain too much noise.

### Choosing the data for the PLS model

**Figure 4:**
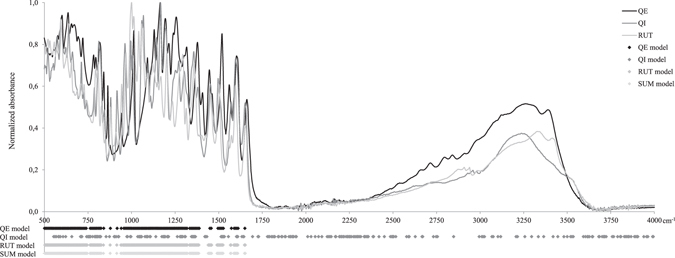
Spectra of quercetin (QE), quercitrin (QI) and rutin (RUT) and marked wavenumbers used in the models presented in Fig. 1.

Our previous work has shown that PLS can build a more useful model if the input data does not contain too large a fraction of non-informative data^16^. The best results were largely obtained with the third technique (Table 1) for which the number of variables was lowered considering only the spectra of standard compounds. However, the technique where spectral data were chosen based on the spectra of the studied compounds and combined with correlation (RS2) produced the largest number of useful models (71 of 186), the technique where only correlation was considered (RS1) produced a medium number of useful models (61 of 186) and the technique where data selection was based only on the spectra of the standard compounds (RS3) produced the smallest number of useful models (54 out of 186). These results show that both aspects can be used in model building and that more research is needed. The spectra of the standards are shown in Fig. 4; this figure also shows which variables were used in the models with the best prediction for quercetin, quercitrin, rutin and the sum of the three flavonoids that are presented on Fig. 1.

### Input data

Two types of models were built regarding the input data and the independent variables. We compared the models built only on spectral data to the models in which variables containing information about the plant species and plant part, where applicable, were also used. In the models that were comparable to HPLC, there was no difference in which technique worked best; however, overall, more useful models were obtained using the additional variables (106 out of all 186 useful models). Most of these models were built on flowers, so the additional input data were the species of the *Fagopyrum* genus. Since the species of the sample is usually known, these data can be added to the input to ease the model prediction; however, most of the important data to predict the concentrations of the studied flavonoids is in the spectral data.

### Number of PLS latent factors

It is difficult to state how many latent factors are most suitable, since the robustness of the models with respect to this number was not tested. Most of the useful and HPLC-comparable models used three latent factors; in general, most of the models used fewer than ten. However, two of the HPLC-comparable models used 11 latent factors. The number of latent factors was chosen using cross-validation, which is a commonly used procedure.

The HPLC is an analytical method of choice in plant material analysis. It is sensitive and robust, it enables simultaneous quantification and separation of many different chemical species. However the extract of the plant material must be prepared first. Here the use of organic solvents can rarely be avoided, also a good efficiency of extraction has to be assured. The time of the analysis of the extract with HPLC is in an hour domain and also uses organic solvents. For in-process control in pharmaceutical, agricultural and food industry there is a need for a fast, reliable and affordable method. The infrared spectroscopy is a good alternative, it is fast, not expensive, and, using a good chemometrical model, the result can be obtained in less than a minute^10^. In a series of samples the time for changing the sample is very short, the cleaning of the surface between two measurements is very simple, using a small towel or a soft brush. It can also be applied without any sample preparation, no solvents are used and there is no destruction of the sample. Infrared spectroscopy is already a part of in-line quality control in the industry^11^. Since there is no destruction of the sample it can also be applied in chemical studies in agriculture since the sample can be further analyzed with other methods.

In this study, we showed that ATR FT-IR can be used for rutin, quercetin and quercitrin determination. Some models give prediction results with accuracy comparable to HPLC analysis. These can be used in cases where precise information about the content of these substances is needed, for example, in the pharmaceutical industry. However, a great number of the models predict flavonoid concentrations more approximately and can therefore be used in the food industry for quality control.

The results revealed a part of the puzzle about the resolution of the spectra that is needed in such complex sample analyses. A higher resolution, 4 cm^−1^, contains more noise but also important information needed to develop a good model. By using a lower resolution, 16 cm^−1^, an appropriate spectral pretreatment method must be applied to obtain a good prediction model.

Ground samples were shown to give models with more accurate results. A reference method that would give information about the concentrations of flavonoids in a small fraction of a sample, from which the spectra is collected when using whole samples, is not available. Therefore, the concentrations determined with the HPLC reference method used are more representative of the information obtained in the ground sample spectra. This could partly explain why the method using ground samples was shown to be more appropriate.

A great part of the research in the field of FT-IR focuses on determining which parts of the spectra are most important and which functional groups are relevant for model building. Our previous results show^15^ that statistical analysis of spectra gives better results compared to selecting specific functional group regions in the case of plant species differentiation. In this study, the best models were obtained by decreasing the amount of spectral data considering the absorption bands of the studied compounds. The highest number of useful models was obtained using a technique that also included correlation computation. IR spectra in general show wide absorption bands of functional groups that are widely present in plant material, and it is difficult to claim in advance which individual wavenumbers will play an important role.

## Materials and Methods

### Samples

14 leaf and 13 flower samples (Table 3) of *F. esculentum* cv. Darja were collected between 3.8.2015 and 24.9.2015 at four-day intervals in Gameljne pod Šmarno goro, Slovenia (46°07′09.2″N14°29′51.5″E).

**Table 3:** Number of whole and ground samples of different buckwheat (*Fagopyrum*) species used in the analysis.

*Fagopyrum* species	Whole leaf samples	Ground leaf samples	Whole flower samples	Ground flower samples
*F. esculentum*	14	15	14	14
*F. tartaricum*	5	6	0	0
*F. rotondatum*	1	1	0	0
*F. giganteum*	6	6	1	1
*F. leptopodum*	3	3	0	0
*F. gracilipes*	2	3	1	1
*F. cymosum*	4	5	1	1

Other seeds (Table 3) of different species were sown in a planter on 4.7.2015 in Grosuplje, Slovenia (45°57′48.3″N14°39′37.3″E). The leaves were collected on 18.8.2015, 1.9.2015 and 3.10.2015. The flowers were collected during their flowering peak in August, September or October 2015.

The seeds were kindly provided by Prof. Dr. Zlata Luthar from the Seed Bank Collection at the Biotechnical faculty of the University of Ljubljana.

### Chemicals

The extraction solvent was of p.a.-grade purity: acetone (JT Baker, Deventer). The solvents used for HPLC analysis were of HPLC-grade purity: water (JT Baker, Deventer), acetonitrile (JT Baker, Deventer), and trifluoroacetic acid (Roth, Karlsruhe). Rutin (Roth, Karlsruhe), quercetin (Kemika, Zagreb) and quercitrin (Roth, Karlsruhe) were used for the calibration curves.

### Extraction method

Dry plant material was ground in an IKA model A10B grinder. If there was a very small amount of available plant material, it was ground using a 2 mm thick metal grinder attached to a Brüder Mannesmann Center Line 130 W model Hobby Tool kit. The samples were macerated in acetone:water (9:1, v/v) to achieve a drug concentration of 10 mg/mL (in most cases, approximately 400 mg of ground drug was macerated in 40 mL of the solvent or 10 mg in 1 mL of the solvent). Maceration lasted for 24 h, including initial and concluding 120 min periods of sonication at 60 °C. The maceration mixtures were placed on window sills for daylight exposure to convert the protofagopyrins to fagopyrins, which were analysed as part of another study. The samples were filtered through 0.45 micron filters prior to analysis.

### Calibration curves

Calibration curves were prepared from the stock solutions in acetone:water (9:1, v/v). The quercetin stock solution was prepared at a concentration of 0.2 mg/mL and was diluted for the calibration curve to concentrations of 0.15, 0.1, 0.05, 0.025, 0.0125, and 0.0025 mg/mL; *R* ^2^ = 0.997. The stock solution for quercitrin was at 0.2 mg/mL and was diluted to 0.15, 0.1, 0.05, 0.0025, 0.00125, 0.0001, and 0.000012 mg/mL; *R* ^2^ = 1.000. The rutin stock solution was prepared in methanol at a concentration of 2.0 mg/mL and was diluted to 1.5, 1.0, 0.6, 0.3, 0.15, and 0.03 mg/mL; *R* ^2^ = 0.997.

### HPLC

The HPLC system (Shimadzu Prominence) consisted of a system controller (CBM-20A), a column oven (CPO-20AC) and a solvent delivery pump with a degasser (DGU-20A5) connected to a refrigerated autosampler (SIL-20AC) with a photodiode array (PDA) detector (SPD-M20A) at 254 nm. The responses of the detectors were recorded using LC Solution software version 1.24 SP1.

Chromatography was performed at 40 °C and a flow rate of 2 mL/min using a Phenomenex Kinetex® XB-C18 column (10 cm × 4.6 mm I.D., 2.7 μm particle size).

The following gradient method using water (solvent A) and acetonitrile (solvent B), both containing 0.1% trifluoroacetic acid, was utilized: 0.01–0.5 min 0% B, 0.5–6.0 min 0–51% B, 6.0–6.01 min 51% B, 6.01–30 min 51–54% B.

### FT-IR

Dried plant material was used for all analyses. The analyses were performed separately on different plant parts: flowers and leaves and on combined data. Spectra were collected from whole and ground samples.

FT-IR spectra were collected using a diamond attenuated total reflection (ATR) accessory from Dura SamplIR Technologies on a Nicolet Instrument Co spectrometer using a DTGS detector. The spectrometer was linked to a computer equipped with Omnic E.S.P. 5.2 software to allow the automated collection of IR spectra.

Each spectrum was collected as an average of 50 scans between 500 and 4000 cm^−1^. Spectra were collected at three resolutions: 4 cm^−1^, 8 cm^−1^ and 16 cm^−1^. Three spectra at each resolution were collected for each sample from different parts of the bulk material. A new background was collected before the three measurements of one sample at each resolution because of the CO_2_ peak. The spectra of the rutin, quercetin and quercitrin standards were collected at a resolution of 4 cm^−1^. For data analysis and model building, all three spectra of one sample were used separately or averaged.

### Data processing

Spectral data analyses were performed in Octave 4.0.0. Using all possible combinations of the 9 parameters, 6912 models were built (listed in a header of Table 1). Models were built using the three separate spectra of each sample and compared to models built using the averaged spectra of each sample. Different spectral pretreatment techniques were used: derivative, standard normal variate (SNV), derivative of SNV, Haar wavelet transform and the derivative of data obtained with the Haar wavelet transform. Wavelet transform yields two data sets, the approximate coefficients and the detailed coefficients; both were compared.

Spectra contain a large number of variables; different methods were used to lower the number of these variables to ¼ of the spectral data before applying partial least squares (PLS). In the first case (RS1), these variables were selected using a Pearson correlation, where ¼ of all spectral variables were chosen based on the largest absolute correlation with the dependent variable for which the model was being built. Another technique (RS2) was to first cut the number of spectral variables in half, taking in the parts of the spectra where the spectra of the standards show most of the absorption peaks (in this case, between 500–1700 cm^−1^ and between 2950–3500 cm^−1^) and then applying Pearson correlation to obtain ¼ of all spectral variables, similarly to the previous case. In addition, the third technique (RS3) to lower the number of variables was selecting ¼ of the spectral variables based on normalized infrared spectra of the rutin, quercetin and quercitrin standards. A preset criterion was the total number of variables (one quarter of the whole spectra), and at least one of the signals of the spectra of the standards had to be higher than the suitable limit (approximately 0.4).

All samples were divided into calibration and validation sets. In the cases where three spectra of the same sample were included in the analysis, all three were assigned to the same set. To obtain the most appropriate number of latent factors for PLS, the leave-one-out method was used on the calibration set to test for the use of 1–20 latent factors. Most commonly, three latent factors were determined to be optimal. We also tested the influence of adding some new independent variables to the spectral data. These were information about the sample that are usually known, such as plant part (leaf or flower) and *Fagopyrum* species. These were added in the form of dummy variables.

The quality of the models was assessed using the correlation *R* of the calibration and validation set of samples and the root mean square error of calibration (RMSEC) and root mean square error of validation (RMSEV).

### Data availability

Infrared spectra analysed during the current study are available from the corresponding author on reasonable request.
